# Supplementary material for: Characterization of N-Terminal Asparagine Deamidation and Clipping of a Monoclonal Antibody
Source: Antibodies (Basel). 2023 Sep 19;12(3):59. doi: 10.3390/antib12030059 (PMC10525203; doi:10.3390/antib12030059)
Supplement: Supplementary file 1 [file antibodies-12-00059-s001.zip › antibodies-2586842-supplementary.pdf]

SUPPLEMENTARY INFORMATION

Table of Contents

1.1 Cation Exchange Data of Heat Stressed Samples 2
1.2 cIEF Data of Heat Stressed Sample 2
1.3 Intact Mass Analysis 3
1.4 Fractioning on Cation Exchange Chromatography 6
1.5 Aggregation Level Comparison 6

1.1 Cation Exchange Data of Heat Stressed Samples

Table S1 Summarized CEX Data of Heat Stressed Samples

Condition	Acidic	Main	Basic
pH7.4 T0	18.0	80.2	1.8
pH7.4 1M25C	24.0	74.5	1.5
pH7.4 2W40C	48.1	31.4	20.5
pH7.4 1M40C	64.8	15.8	19.4
pH6 T0	19.2	78.4	2.4
pH6 1M25C	24.6	72.8	2.5
pH6 2W40C	49.9	34.0	16.1
pH6 1M40C	56.5	12.9	30.6

Note: “M” means “month” and “C” means “Celsius degree”.

1.2 cIEF Data of Heat Stressed Sample

Table S2 Summarized cIEF Data of Heat Stressed Samples

Condition	Acidic	Main	Basic
pH7.4 T0	29.15	66.44	4.40
pH7.4 1M25C	33.53	62.44	4.03
pH7.4 2W40C	57.99	37.98	4.04
pH7.4 1M40C	75.17	21.25	3.56
pH6 T0	28.48	67.53	4.00
pH6 1M25C	33.63	62.81	3.56
pH6 2W40C	59.13	37.11	3.75
pH6 1M40C	77.68	19.60	2.74

Note: “M” means “month” and “C” means “Celsius degree”.

1.3 Intact Mass Analysis

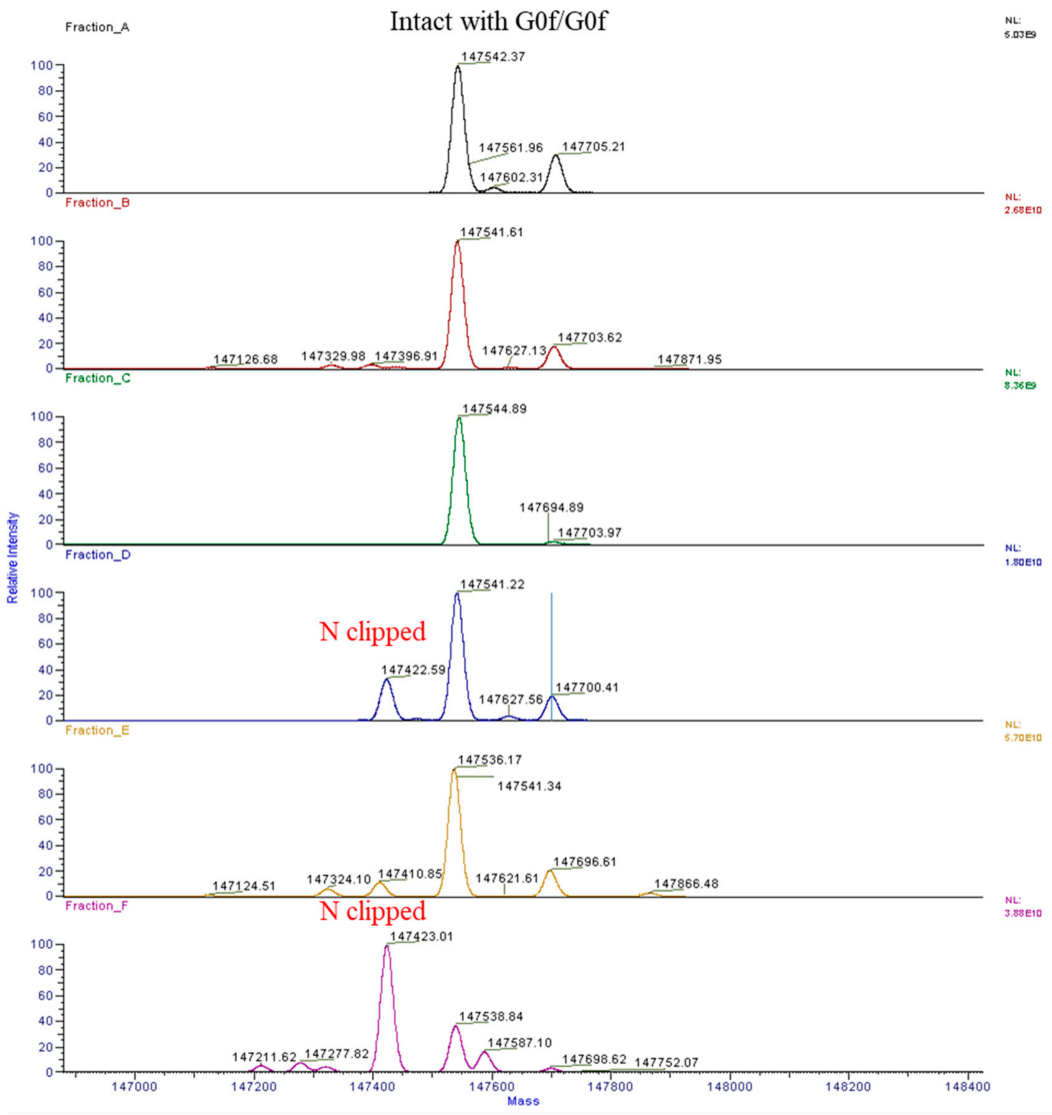

Figure S1 **Intact Mass of CEX fractions**

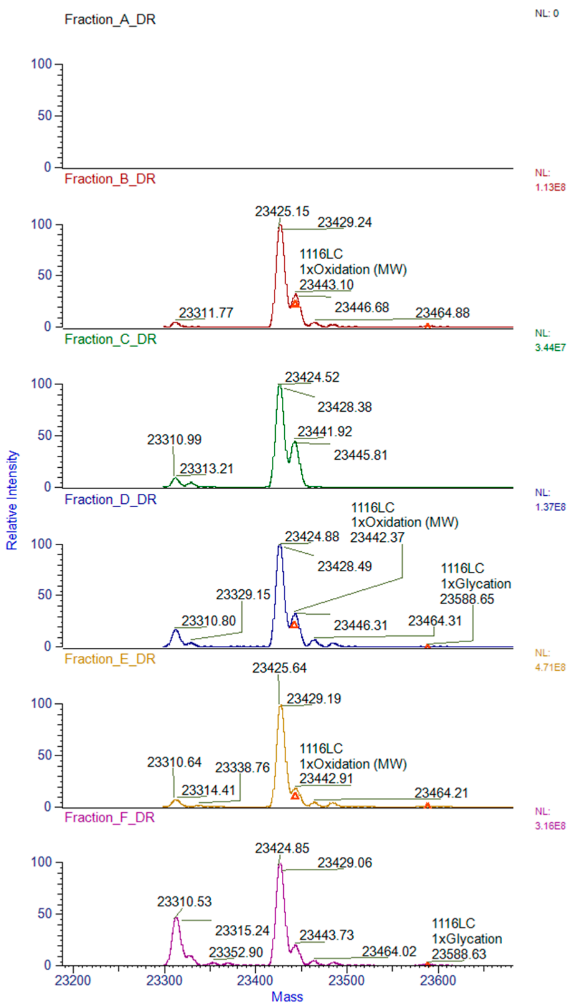

Figure S2 **Reduced Light Chain Mass of CEX fractions**

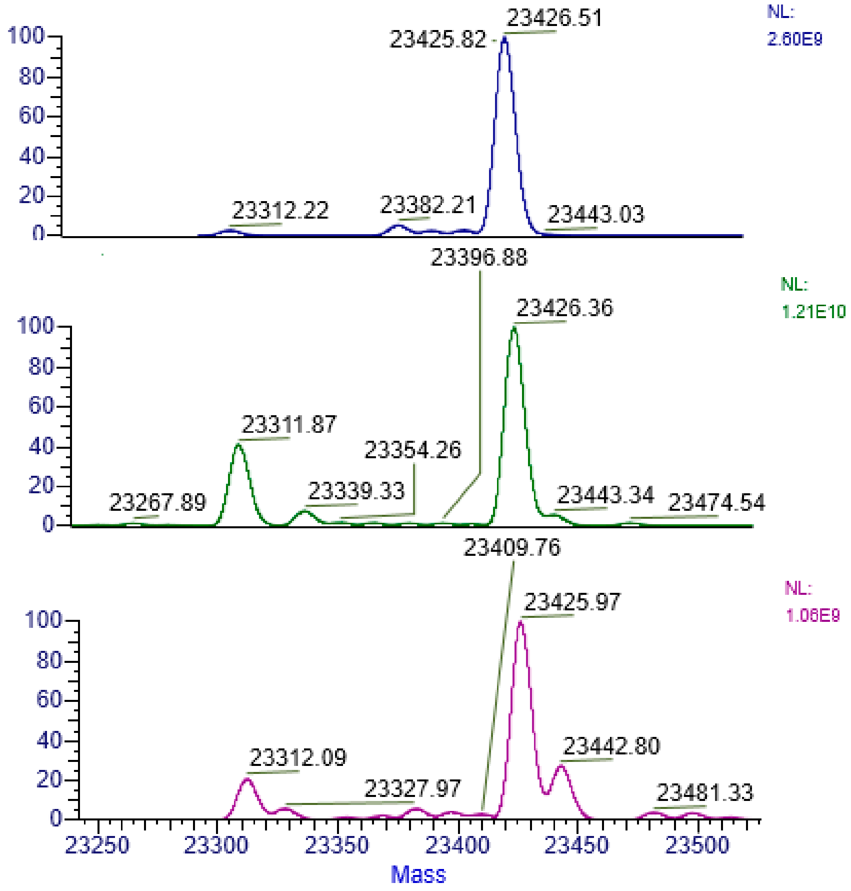

Figure S3 Clipping comparison between Drug Substance in final formulation and clarified medium by reduced intact mass analysis. (Top) Reference Standard; (Middle) 40C, 1 month sample in final formulation; (Bottom) 40C, 2 weeks sample in clarified medium.

1.4 Fractioning on Cation Exchange Chromatography

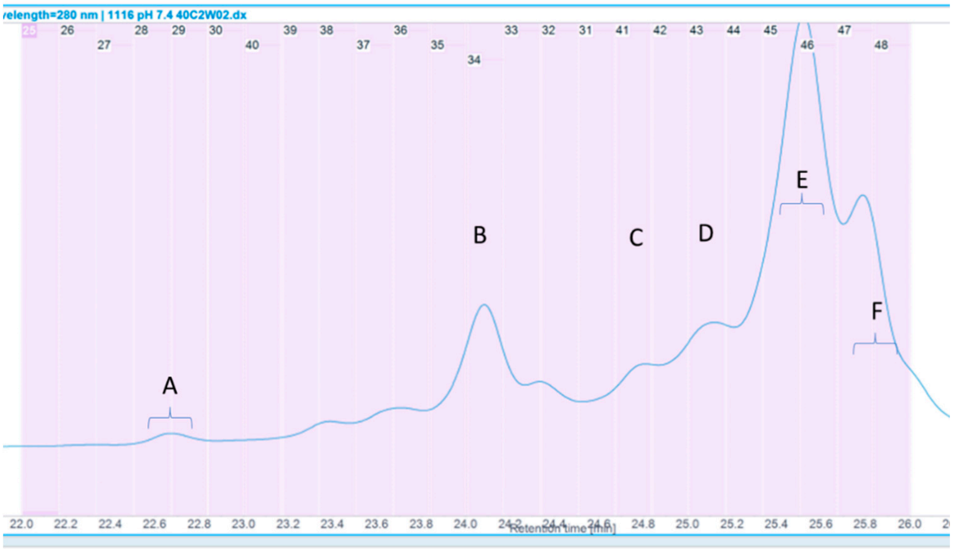

Figure S4 CEX fractionation

1.5 Aggregation Level Comparison

Table S3 Aggregation Level Comparison between different IgG molecules

	**Aggregates level measured by SEC**		
	**mAb1**	**mAb2**	**mAb3**
Control, T0	0.6	0.2	0.7
25°C, 1 m	NA	0.4	0.9
25°C, 3 m	0.8	0.5	1.2
25°C, 6 m	1	0.8	1.7
25°C, 12 m	1.3	NA	NA
40°C, 1 m	1.1	0.8	1.8
40°C, 3 m	1.8	1.5	3.6
40°C, 6 m	2.5	3.5	NA

mAb1 is corresponding to the molecule studied in the manuscript; NA = Not Available
